# Crosstalk Analysis and Performance Evaluation for Torus-Based Optical Networks-on-Chip Using WDM

**DOI:** 10.3390/mi11110985

**Published:** 2020-10-31

**Authors:** Tingting Song, Yiyuan Xie, Yichen Ye, Shujian Wang, Yingxue Du

**Affiliations:** 1 School of Electronic and Information Engineering, Southwest University, No. 2 Tiansheng Road, Beibei District, Chongqing 400715, China; ttsong_53@163.com (T.S.); ycye451@swu.edu.cn (Y.Y.); shujianwangswu@163.com (S.W.); yingxueduswu@163.com (Y.D.); 2 Chongqing Key Laboratory of Nonlinear Circuits and Intelligent Information Processing, No. 2 Tiansheng Road, Beibei District, Chongqing 400715, China

**Keywords:** crosstalk noise, four-wave mixing, network performance, optical networks-on-chip, wavelength division multiplexing

## Abstract

Insertion loss and crosstalk noise will influence network performance severely, especially in optical networks-on-chip (ONoCs) when wavelength division multiplexing (WDM) technology is employed. In this paper, an insertion loss and crosstalk analysis model for WDM-based torus ONoCs is proposed to evaluate the network performance. To demonstrate the feasibility of the proposed methods, numerical simulations of the WDM-based torus ONoCs with optimized crossbar and crux optical routers are presented, and the worst-case link and network scalability are also revealed. The numerical simulation results demonstrate that the scale of the WDM-based torus ONoCs with the crux optical router can reach 6 × 5 or 5 × 6 before the noise power exceeds the signal power, and the network scale is 5 × 4 in the worst case when the optimized crossbar router is employed. Additionally, the simulated results of OptiSystem reveal that WDM-based torus ONoCs have better signal transmission quality when using the crux optical router, which is consistent with previous numerical simulations. Furthermore, compared with the single-wavelength network, WDM-based ONoCs have a great performance improvement in end-to-end (ETE) delay and throughput according to the simulated results of OPNET. The proposed network analysis method provides a reliable theoretical basis and technical support for the design and performance optimization of ONoCs.

## 1. Introduction

With the rapid development of manufacturing processes, on-chip devices have been manufactured in nano-layers. Integrating hundreds of millions of components on a single chip has become a reality and the number of components has continued increasing, so the multiprocessor systems-on-chip (MPSoCs) has become the mainstream of on-chip designs [1,2,3]. With the growing requirements of chip computing performance and multitasking simultaneous processing capability, electrical networks-on-chip (ENoCs) cannot meet the needs of MPSoCs development due to the time delay, bandwidth, and power consumption problems [4]. Optical networks-on-chip (ONoCs), which can break through these bottlenecks, have been proven an effective method to solve the problems faced by ENoCs [5,6,7,8,9]. ONoCs combine both the characteristics of the electrical interconnect layer, which implements arbitration control, and the optical layer to transfer data [10,11,12]. However, due to the demand of big data applications and very-large-scale integration, the single-wavelength data transmission in ONoCs no longer meets the bandwidth requirements for large-scale communications. Therefore, wavelength division multiplexing (WDM) technology, in which multiple optical signals are transmitted in a single waveguide simultaneously, is introduced into the ONoC to effectively improve the bandwidth [13,14]. At present, WDM-based ONoCs have great application prospects in the fields of big data centers, multi-core systems, and high-speed communications. The continuous innovation of silicon-based optical device structures and continuous improvement in comprehensive performance have directly promoted the WDM-based ONoCs as the main design mode of on-chip networks.

It has been demonstrated that the network can benefit from high bandwidth and low latency according to some research on WDM-based ONoCs [15,16,17]. Although the WDM technology employed has improved the network performance of ONoCs greatly, the insertion loss and intrinsic crosstalk noise caused by photonic devices are still unavoidable [18]. It has been revealed that these inevitable factors will cause the severe performance degradation of photonic networks [19,20,21]. Besides, for WDM-based ONoCs, the nonlinear effects cannot be removed or ignored, especially the four-wave mixing (FWM) phenomenon that will produce a prodigious proportion of nonlinear crosstalk noise [22,23,24]. The accumulation of crosstalk noise is an important factor that leads to signal distortion, optical signal-to-noise ratio (OSNR) attenuation, and network performance degradation. Ultimately, the scalability of WDM-based ONoCs is greatly restricted.

Nowadays, the research on crosstalk characteristics of ONoCs with single-wavelength is very mature. For example, complete noise analysis models are established to explore and analyze the performance of the worst case for various interconnection topologies, such as mesh-based, torus-based, fat-tree-based, and ring-based ONoCs [19,20,21,25]. The presented analytical models for different networks are performed hierarchically at basic-devices, routers, and networks levels. Recently, this mature modeling method was considered for the network performance evaluation in other works, such as ONoCs with a low-power thermally resilient and fault-tolerant routing mechanism [26,27]. It is noted that the analytical models in the above research are based on the traditional ONoCs with single-wavelength, which does not consider the influence of the intra-channel crosstalk and nonlinear noise existing in WDM-based ONoCs, and they are not suitable for the performance analysis of ONoCs employing WDM technology. Fortunately, a formal study model of the crosstalk characteristic for WDM-based ONoCs was proposed immediately [15]. However, the previous research on crosstalk characteristics in WDM-based ONoCs almost only focused on original linear crosstalk noise and ignored the nonlinear noise generated by the nonlinear effects. Our previous research demonstrated that the noise produced by the nonlinear FWM effect is a non-negligible issue for the performance evaluation of WDM-based ONoCs, but the analytical model presented in that research is based on mesh network topology [16]. Compared with mesh topology, the torus network has topological advantages in terms of network diameter, average hop distance, and better connectivity [28]. It can obtain less communication latency and higher saturation throughput under the same network scale [29,30].

Therefore, considering the advantages of torus topology and its commercial application [31,32], a performance analysis model of the insertion loss and crosstalk calculation method for torus-based ONoCs with WDM is proposed and analyzed systematically in this paper. The WDM-based optimized crossbar (WOPC) optical router and WDM-based Crux (WCX) optical router are adopted to quantify the analytical model. In the numerical simulation, the performance of the first, second, and third longest links with eight optical wavelengths is evaluated to find the worst-case link and maximum network size. Furthermore, an optical transmission system for the presented WDM-based torus ONoCs is established based on OptiSystem 17.0 (developed by Optiwave Systems Inc., Ottawa, ON, Canada) to evaluate communication quality. Simulation results show that crosstalk noise degenerates the OSNR performance of WDM-based ONoCs severely, results in optical signal distortion, and eventually limits network scalability. For instance, when a WCX optical router is employed and the scale of the network is 4 × 4, input power is 0 dBm in the worst case, the average OSNR of eight optical signals is 8.8 dB, and the values of linear crosstalk and nonlinear power are −38.4 and −37.6 dBm, respectively. If a WOPC optical router is used under the same conditions, the corresponding values of these indicators are 1.6 dB, −36.3 dBm, and −37.6 dBm, respectively. It can be seen that the nonlinear FWM plays a significant role in the performance degradation of WDM-based ONoCs, and the network can achieve a better performance when using the WCX optical router. Additionally, the network performances such as end-to-end (ETE) delay and throughput are also simulated by OPNET. It can be obtained from simulation results that WDM-based ONoCs have a great performance improvement in throughput and ETE delay compared with the single-wavelength network at the cost of a more serious restriction of network scalability induced by crosstalk noise.

## 2. Performance Analysis Model for WDM-Based Torus ONoCs

### 2.1. WDM-Based Basic Photonic Devices

**Figure 1:**
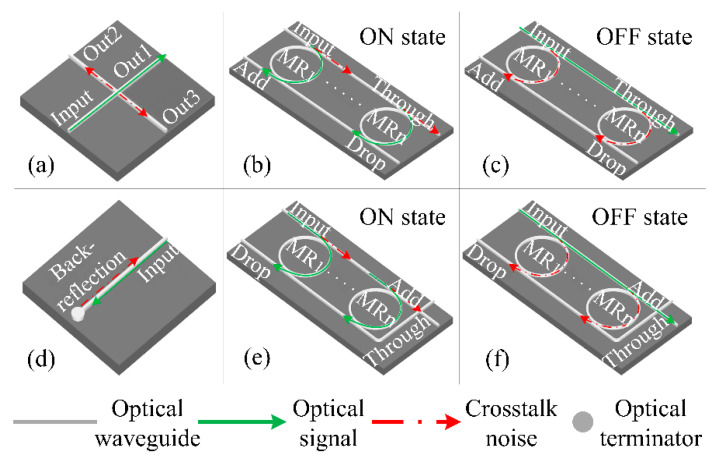
(**a**) Waveguide crossing; (**b**) wavelength division multiplexing (WDM)-based optical parallel switching elements (WPSE) at ON state; (**c**) WPSE at OFF state; (**d**) optical terminator; (**e**) WDM-based optical crossing switching elements (WCSE) at ON state; (**f**) WCSE at OFF state.

In this section, we analyze and model the power loss and crosstalk noise at the basic photonic device level. Silicon-based waveguides, waveguide crossings, and micro-resonators (MRs) are three components of basic optical switching elements (BOSEs). The switching time of each MR is just 30 ps and the diameter is less than 10 μm [33], which is suitable for MPSoCs. The WDM-based optical parallel and crossing switching elements (WPSE and WCSE, respectively) are shown in Figure 1. They are essential parts of the optical router. With the help of WPSE and WCSE, the path of propagation of the optical signal can be adjusted. The routing of optical signals cannot be accomplished without these devices. Both WPSE and WCSE have an OFF state and ON state, and the trend of optical signal and crosstalk noise is indicated with different colors. Each MR has a certain resonance wavelength *λ_MR_*. When the MR is powered in the OFF state, the wavelength of the optical signal *λ_s_* does not satisfy the resonance condition of the MR, that is, *λ_s_* ≠ *λ_MR_*, and the optical signal passes through the MR to the Through port, as shown in Figure 1c,f. On the contrary, when *λ_s_* = *λ_MR_* (i.e., ON state), the input optical signal couples into MR and propagates to the Drop port, as shown in Figure 1b,e. The different states can be controlled by changing the voltage applied to the MRs [34].

As shown in Figure 1a, waveguide crossing, which is inherently required in ONoCs, is a structure of the intersection of two waveguides. The crosstalk of a waveguide crossing mainly results from the strong diffraction of the guiding modes when they transmit through the crossing region. The output power of the outputs (Out1, Out2, and Out3) can be calculated by Equation (1) to (3), while the optical signal power is *P_in_* and wavelength is *λ_s_n_*.

(1) $$P_{O1}^{\lambda_{s\_n}}=P_{in}^{\lambda_{s\_n}}\cdot L_{c}$$

(2) $$P_{O2}^{\lambda_{s\_n}}=P_{in}^{\lambda_{s\_n}}\cdot K_{11}$$

(3) $$P_{O3}^{\lambda_{s\_n}}=P_{in}^{\lambda_{s\_n}}\cdot K_{12}$$

where *L_c_* is the insertion loss when the optical signal travels through each waveguide crossing. *K*_11_ and *K*_12_ are crossing crosstalk coefficients that can evaluate the output power from the other two ports when the optical signal transmits through the waveguide crossing. The values of *K*_11_ and *K*_12_ are equal when the crossing angle is 90°, and they can be smaller than −40 dB [35].

The WPSE at the ON and OFF states is shown in Figure 1b,c, respectively. If the optical signal power on the input port is *P_in_*, the output powers at the Through and Drop port of different states can be expressed as:

(4) $$P_{T_{P\_OFF}}^{\lambda_{s\_n}}=P_{in}^{\lambda_{s\_n}}\cdot \prod_{i=1}^{N}L_{p1}^{\lambda_{s\_i}}$$

(5) $$P_{D_{P\_OFF}}^{\lambda_{s\_n}}=P_{in}^{\lambda_{s\_n}}\cdot \prod_{i=1}^{n-1}{\left(L_{p1}^{\lambda_{s\_i}}\right)}^{2}\cdot \sum_{j=1}^{N}K_{\lambda_{s\_n,\;off}}^{\lambda_{s\_j}}$$

(6) $$P_{T_{P\_ON}}^{\lambda_{s\_n}}=P_{in}^{\lambda_{s\_n}}\cdot K_{\lambda_{s\_n,on}}^{\lambda_{s\_n}}\cdot \prod_{i=1,\;i\neq n}^{N}L_{p1}^{\lambda_{s\_i}}$$

(7) $$P_{D_{P\_ON}}^{\lambda_{s\_n}}=P_{in}^{\lambda_{s\_n}}\cdot \prod_{i=1}^{n-1}{\left(L_{p1}^{\lambda_{s\_i}}\right)}^{2}\cdot \left(L_{p2}^{\lambda_{s\_n}}+\sum_{j=1,\;j\neq n}^{N}K_{\lambda_{s\_n,\;on}}^{\lambda_{s\_j}}\right)$$

Figure 1d is the optical terminator whose function is to absorb the optical signal and avoid its back-reflection. The WCSE at the ON and OFF states are shown in Figure 1e,f, respectively. Similarly, for the WCSE, the output power of the Through and Drop port can be calculated as:

(8) $$P_{T_{C\_OFF}}^{\lambda_{s\_n}}=P_{in}^{\lambda_{s\_n}}\cdot \prod_{i=1}^{N}L_{p1}^{\lambda_{s\_i}}\cdot L_{c}$$

(9) $$P_{D_{C\_OFF}}^{\lambda_{s\_n}}=P_{in}^{\lambda_{s\_n}}\cdot \prod_{i=1}^{n-1}{\left(L_{p1}^{\lambda_{s\_i}}\right)}^{2}\cdot \left(\sum_{j=1}^{N}K_{\lambda_{s\_n,\;off}}^{\lambda_{s\_j}}+K_{12}\prod_{i=1}^{N}{\left(L_{p1}^{\lambda_{s\_i}}\right)}^{2}\right)$$

(10) $$P_{T_{C\_ON}}^{\lambda_{s\_n}}=P_{in}^{\lambda_{s\_n}}\cdot (\prod_{i=1,\;i\neq n}^{N}L_{p1}^{\lambda_{s\_i}})\cdot L_{c}\cdot K_{\lambda_{s\_n,\;on}}^{\lambda_{s\_n}}$$

(11) $$P_{D_{C\_ON}}^{\lambda_{s\_n}}=P_{in}^{\lambda_{s\_n}}\cdot \prod_{i=1}^{n-1}{\left(L_{p1}^{\lambda_{s\_i}}\right)}^{2}\cdot \left(L_{p2}^{\lambda_{s\_n}}+\sum_{j=1,\;j\neq n}^{N}K_{\lambda_{s\_n,on}}^{\lambda_{s\_j}}\right)+P_{in}^{\lambda_{s\_n}}\cdot {\left(K_{\lambda_{s\_n,on}}^{\lambda_{s\_n}}\cdot \prod_{i=1,\;i\neq n}^{N}L_{p1}^{\lambda_{s\_i}}\right)}^{2}\cdot K_{12}$$

In the formulas above, $L_{p1}^{\lambda_{s\_i}}$ (i=1, 2,⋯N) is the insertion loss coefficient of the optical signal *λ*_s_n_ through the MR, whose resonant wavelength is *λ*_s_i_, and $L_{p2}^{\lambda_{s\_n}}$ is the insertion loss coefficient corresponding to optical signal *λ*_s_n_ coupled into MR_n_. *K* is the crosstalk coefficient, which is generated by the optical signals through the different MRs in the OFF or ON state.

### 2.2. General Optical Router Model

**Figure 2:**
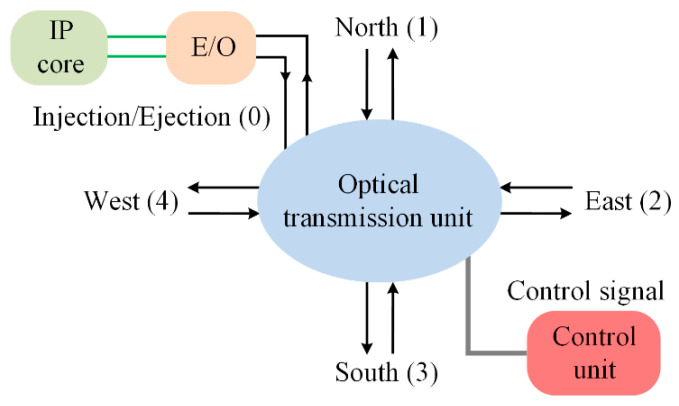
General 5 × 5 optical router model.

The optical router is the key component in WDM-based ONoCs. The main function of an optical router is to realize data routing and exchanging between two IP cores, and optical signals in an optical router cannot overlap. The basic 5 × 5 optical router model that we used in this paper is shown in Figure 2. The five bidirectional ports are named Injection/Ejection, North, South, West, and East, and they are represented numerically by 0, 1, 2, 3, and 4, respectively. The Injection/Ejection port connects with the IP core through the electronic optical (E-O) and optical-electronic (O-E) interface, which accomplish the conversion between optical and electrical signals. The electric control unit is used for the optical path command.

Considering the optical signal *λ_s_n_* travels in the optical router from the input port *i* to output port *j*, the output power can be calculated as follows:

(12) $$P_{ij}^{\lambda_{s\_n}}=P_{in}^{\lambda_{s\_n}}\cdot L_{ij}^{R}$$

(13) $$L_{ij}^{R}=L_{b}^{k_{1}}L_{P\_ON}^{k_{2}}L_{P\_OFF}^{k_{3}}L_{C\_ON}^{k_{4}}L_{C\_OFF}^{k_{5}}L_{c}^{k_{6}}L_{tras}$$

(14) $$L_{trans}=10^{-\alpha L/10}$$

where i, j∈(0,1,2,3,4)  and  i≠j. $L_{ij}^{R}$ is the total insertion loss at the destination port of optical router R, which can be calculated by Equation (13). In Equation (13), *L_b_* represents the insertion loss when the optical signal goes through a bending waveguide, *L_P/C_ON/OFF_* is the loss coefficient when the signal traverses a WPSE/WCSE at the ON/OFF state, and superscript *k* is the number of bending waveguides, WPSE/WCSE at the ON/OFF state, and waveguide crossings in the optical transmission link. In Equation (6), the transmission loss is denoted by *L_trans_*, in which *α* is the waveguide attenuation coefficient and *L* is the optical transmission length [36].

### 2.3. Nonlinear FWM Crosstalk Noise Analysis

The four-wave mixing (FWM) phenomenon [37,38] caused by the third-order nonlinear effect is where a new optical wave is produced by the interaction between coherent signal light and incoherent pump light in a highly nonlinear fiber. When the wavelength of the newly generated optical wave is located in the position of the original optical signals, its power will be converted into crosstalk noise, which is named nonlinear FWM crosstalk noise.

**Figure 3:**
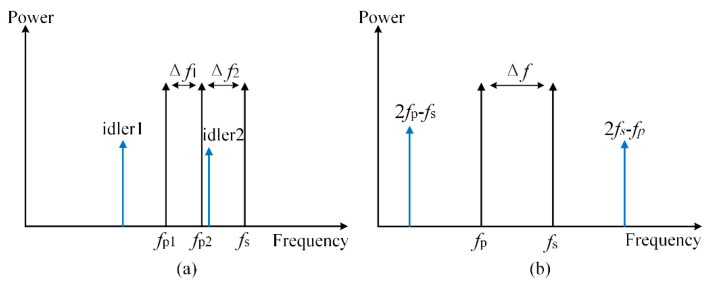
(**a**) The process of four-wave mixing (FWM); (**b**) degenerated FWM.

The process of FWM is shown in Figure 3a, and the newly generated optical wave in frequency *f_i_*_1_ (idler1) and *f_i_*_2_ (idler2) can be expressed as:

(15) $$f_{i1}=f_{p1}+f_{p2}-f_{s}$$

(16) $$f_{i2}=f_{p1}-f_{p2}+f_{s}$$

Moreover, when *f_p_*_1_ = *f_p_*_2_ = *f_p_*, the new optical wave will emerge at frequency *f_idler_* = 2*f_p_* − *f_s_*, and this phenomenon is named degenerated four-wave mixing (DFWM) [39], as shown in Figure 3b. Based on the previous research [40], the power of the newly generated optical wave is given as:

(17) $$P_{FWM}=\frac{4}{27}\cdot (\frac{\gamma P_{p}}{\alpha })\cdot P_{s}\cdot \eta$$

where *P*_p_ and *P*_s_ are the power of the input optical signals at frequencies *f*_p_ and *f*_s_. *γ*, *α*, and *η* are the waveguide nonlinear coefficient, attenuation coefficient, and FWM efficiency, respectively. The expressions of *γ* and *η* are

(18) $$\gamma =\frac{2\pi n_{2}}{\lambda_{s}A_{eff}}$$

(19) $$\eta =\frac{\alpha^{2}}{\alpha^{2}+{\left(\Delta \beta \right)}^{2}}\left(1+4\frac{e^{-\alpha L}}{{\left(1-e^{-\alpha L}\right)}^{2}}\cdot \sin^{2}(\Delta \beta \cdot \frac{L}{2})\right)$$

In Formulas (18) and (19), *n*_2_ and *A_eff_* are the nonlinear refractive index and effective core area in the silicon-based waveguide, respectively. ∆*β* and *L* are the propagation constant difference and the length of the optical transmission link. ∆*β* can be given as:

(20) $$\Delta \beta =\frac{2\pi \lambda_{s}^{2}}{c}\cdot \Delta f^{2}\cdot \left(D(\lambda )+\Delta f\cdot \frac{\lambda_{s}^{2}}{c}\cdot \frac{dD}{d\lambda }\right)$$

where *∆f* is the frequency separation between *fp* and *fs*. *c* is the speed of light in vacuum. *D*(*λ*) denotes the waveguide chromatic dispersion and *dD*/*dλ* is the dispersion slope [41].

According to the analysis above, when multiple optical wave signals are transmitted in the optical link, the accumulated nonlinear FWM crosstalk on the optical signal λ*_i_* can be calculated by the following formulas:

(21) $$P_{C\_FWM}(2f_{p}-f_{s})=\sum_{L}\sum_{f_{p}}\sum_{f_{s}}P_{FWM}(f_{p},f_{s})$$

(22) $$P_{C\_FWM}(\lambda_{i})=\sum_{j}\sum_{k}P_{FWM}(\lambda_{j},\lambda_{k})\left\{2j-k=i\right\}$$

### 2.4. Analysis Model of WDM-Based Torus ONoCs

**Figure 4:**
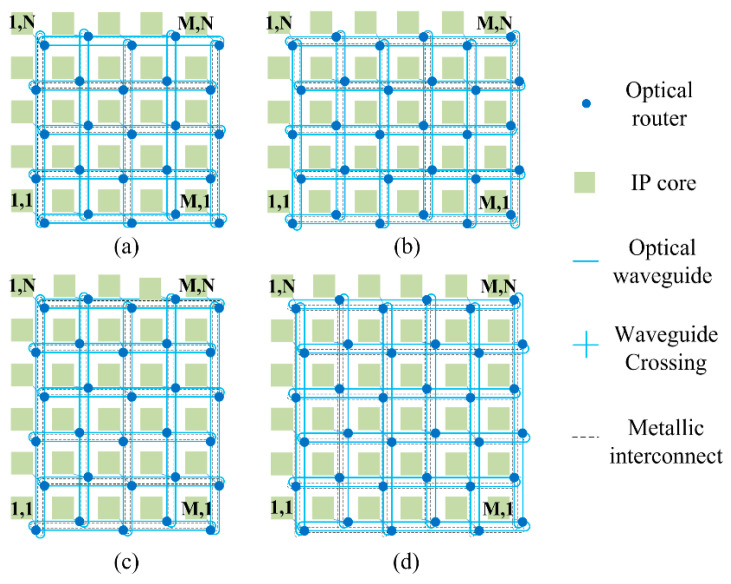
WDM-based torus optical networks-on-chip (ONoCs): (**a**) M and N are both odd numbers; (**b**) M is an even number and N is an odd number; (**c**) M is an odd number and *N* is an even number; (**d**) M and N are both even numbers.

In this part, we systematically analyze and model the power loss, crosstalk noise, OSNR, and BER bit error ratio (BER) for WDM-based torus ONoCs. For *M × N* WDM-based torus ONoCs, we can divide it into four structures according to the even or odd values of M and N, as shown in Figure 4. Annular passages are used in the horizontal and vertical directions of the network. Thus, the optical routers that are located in each line and column are dispersed from one waveguide to two. This structure greatly reduces the number of routers in signal transmission links. Thence, torus-based ONoCs have better network performance due to the introduction of less crosstalk noise and effective reduction in power consumption.

On the basis of different torus-based architectures, we put forward different calculation models. The main performance indicators we take into consideration are insertion loss, linear and nonlinear crosstalk noise, OSNR and BER. In our analytical model, the optical signals transmission rule follows the XY routing algorithm, in which optical signals can only be transmitted from the X (horizontal) direction to Y (vertical) direction. In this paper, our performance analysis model is based on the first, second, and third longest optical links to find the worst case. According to the XY routing algorithm, each optical link that we analyzed has four candidate links. For example, the first longest links includes (M, 1) to (1, N), (M, N) to (1, 1), (1, 1) to (M, N), and (1, N) to (M, 1). The worst-case optical links under different network size determine the network scalability.

According to the general model, the output power for optical signal *λ_s_n_* transmitted from the core (*x_0_*, *y_0_*) to (*x_1_*, *y_1_*) can be calculated by the following equation.

(23) $$P_{\left(x_{0},y_{0}),(x_{1},y_{1}\right)}^{\lambda_{s\_n}}=P_{in}^{\lambda_{s\_n}}\cdot L_{\left(x_{0},y_{0}),(x_{1},y_{1}\right)}^{\lambda_{s\_n}}\cdot 10^{-\frac{\alpha \left[(\left|x_{1}-x_{0}\right|)\cdot L_{wd}+(\left|y_{1}-y_{0}\right|)\cdot L_{ht}\right]}{10}}\;$$

where $x_{0},x_{1}\in (1,2\cdots M)\;$ and $y_{0},y_{1}\in (1,2\cdots N)$. $L_{\left(x_{0},y_{0}),(x_{1},y_{1}\right)}^{\lambda_{s\_n}}$ is the insertion loss of the optical signal *λ_s_n_* in the transmission link (*x_0_*, *y_0_*) to (*x_1_*, *y_1_*), which can be calculated based on the analytical model at the optical router level in Section 2.2. The silicon-based waveguide attenuation coefficient α is also described in the previous section. *L_wd_* and *L_ht_* are distances between routers in the horizontal and vertical directions, respectively [16].

The general crosstalk noise model in the optical transmission link (*x*_0_, *y*_0_) to (*x*_1_, *y*_1_) can be expressed as:

(24) $$N_{\left(x_{0},y_{0}),(x_{1},y_{1}\right)}^{\lambda_{s\_n}}=\sum_{\left(x_{0},y_{0}\right)}^{\left(x_{1},y_{1}\right)}N_{\left(x_{i},y_{i}\right)}^{\lambda_{s\_n}}\cdot L_{tras}+N_{FWM}$$

(25) $$N_{\left(x_{i},y_{i}\right)}^{\lambda_{s\_n}}=P_{in}^{\lambda_{s\_n}}\cdot \sum_{i=1}^{4}L_{0i}^{R}\cdot L_{c}^{k}\cdot K_{a,b,m}+N_{s\_i,(i\neq n)}$$

(26) $$N_{s\_i,(i\neq n)}=P_{in}^{\lambda_{s\_n}}{\prod_{i=1}^{n-1}\left(L_{p1}^{\lambda_{s\_i}}\right)}^{2}\cdot \sum_{j=1,j\neq n}^{N}K_{\lambda_{s\_n,on}}^{\lambda_{s\_j}}$$

(27) $$N_{FWM}=\sum_{L}\sum_{\lambda_{p}}\sum_{\lambda_{s}}P_{DFWM}(\lambda_{p},\lambda_{s})$$

in which $N_{\left(x_{i},y_{i}\right)}^{\lambda_{s\_n}}$ is crosstalk noise generated at optical router (*x_i_*, *y_i_*) and *N_FWM_* denotes nonlinear crosstalk noise introduced by the FWM effect. *K_a,b,m_* is the crosstalk noise coefficient, *a* and *b* are signal input and output ports of the optical router, respectively, and *m* is the noise injected port.

When optical signal *λ_s_n_* travels from core (*x_0_*, *y_0_*) to (*x_1_*, *y_1_*), the accumulated crosstalk noise power on *λ_s_n_* can be calculated as

(28) $$P_{N}^{\lambda_{s\_n}}=\sum_{\left(x_{0},y_{0}\right)}^{\left(x_{1},y_{1}\right)}\frac{N_{\left(x_{i},y_{i}\right)}^{\lambda_{s\_n}}\cdot L_{\left(x_{0},y_{0}),(x_{1},y_{1}\right)}^{\lambda_{s\_n}}}{L_{\left(x_{0},y_{0}),(x_{i},y_{i}\right)}^{\lambda_{s\_n}}}\cdot L_{tras}+N_{FWM}$$

Therefore, the OSNR and BER of optical signal *λ_s_n_* at the destination core can be calculated by

(29) $$OSNR^{\lambda_{s\_n}}=10\log\frac{P_{in}^{\lambda_{s\_n}}\cdot L_{\left(x_{0},y_{0}),(x_{1},y_{1}\right)}^{\lambda_{s\_n}}}{P_{N}^{\lambda_{s\_n}}}$$

(30) $$BER^{{}^{\lambda_{s\_n}}}=\frac{1}{2}\left(1-\frac{2}{\sqrt{\pi }}\int_{0}^{\frac{\sqrt{OSNR^{\lambda_{s\_n}}}}{2}}e^{-t^{2}}dt\right)$$

### 2.5. Optical Links Selection in WDM-Based Torus ONoCs

**Figure 5:**
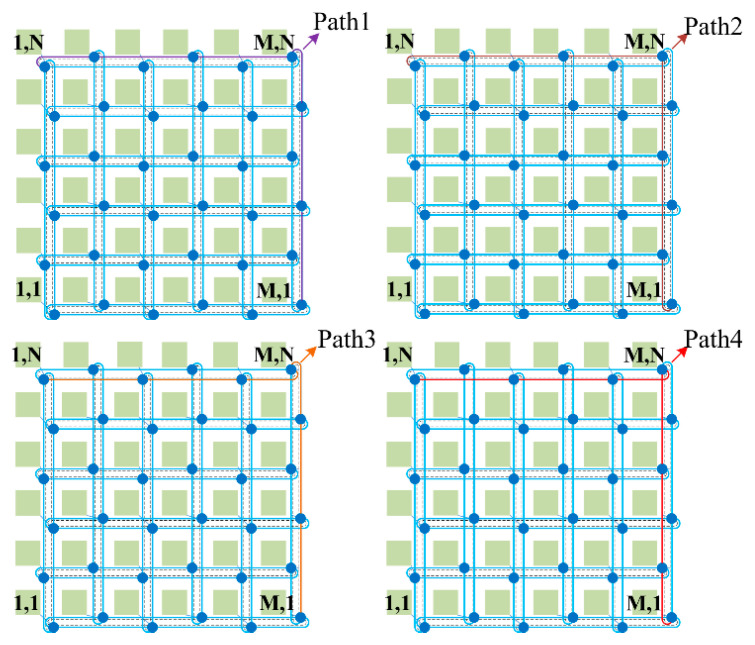
Different paths among the first longest links.

The minimum OSNR optical link has the maximum signal power loss and crosstalk noise, and it determines the scalability of WDM-based torus ONoCs. From the analysis of the optical router, it is easy to know that the first longest link has the maximum number of optical routers and largest transmission loss, but it may not be the optical link that has the largest crosstalk noise introduced. Therefore, we chose the first, second, and third longest optical links to find the worst-case OSNR link. The four different paths of each first longest link are shown in Figure 5, and four candidate links in it are signed with different colors. The output power of optical *λ_s_n_* at the destination core at different longest links is presented below in detail. The first, second, and third longest links we selected for calculation are (1, N) to (M, 1), (1, N) to (M − 1, 1), and (1, N − 1) to (M − 1, 1), respectively.

(31) $$\begin{array}{l} P_{in}^{\lambda_{s\_n}}\cdot L_{\left(x_{0},y_{0}),(x_{1},y_{1}\right)}^{\lambda_{s\_n}}=\;(M,\;N\;both\;even\;numbers)\;\\ \left\{ \begin{array}{l} P_{in}^{\lambda_{s\_n}}\cdot L_{04}^{}\cdot {\left(L_{c}^{2}L_{42}^{}\right)}^{\frac{M-2}{2}}\cdot L_{41}^{}\cdot {\left(L_{c}^{2}L_{13}^{}\right)}^{\frac{N-2}{2}}\cdot L_{10}^{}\cdot L_{tras}^{1},\;(x_{0}=1,y_{0}=N,x_{1}=M,y_{1}=1)\\ P_{in}^{\lambda_{s\_n}}\cdot L_{02}\cdot {\left(L_{c}^{6}L_{42}\right)}^{\frac{M-4}{2}}\cdot L_{c}^{6}\cdot L_{41}\cdot L_{c}^{4}\cdot {\left(L_{c}^{6}L_{13}\right)}^{\frac{N-2}{2}}\cdot L_{10}\cdot L_{tras}^{2},\;(x_{0}=1,y_{0}=N,x_{1}=M-1,y_{1}=1)\\ P_{in}^{\lambda_{s\_n}}\cdot L_{02}\cdot {\left(L_{c}^{6}L_{42}\right)}^{\frac{M-4}{2}}\cdot L_{c}^{6}\cdot L_{43}\cdot {\left(L_{c}^{6}L_{13}\right)}^{\frac{N-4}{2}}\cdot L_{c}^{6}\cdot L_{10}\cdot L_{tras}^{3},\;(x_{0}=1,y_{0}=N-1,x_{1}=M-1,y_{1}=1) \end{array}\right. \end{array}$$

(32) $$\begin{array}{l} P_{in}^{\lambda_{s\_n}}\cdot L_{\left(x_{0},y_{0}),(x_{1},y_{1}\right)}^{\lambda_{s\_n}}=\;(M,\;N\;both\;odd\;numbers)\\ \left\{ \begin{array}{l} P_{in}^{\lambda_{s\_n}}\cdot L_{02}^{}\cdot {\left(L_{c}^{6}L_{42}^{}\right)}^{\frac{M-3}{2}}\cdot L_{c}^{6}\cdot L_{41}^{}\cdot L_{c}^{4}\cdot L_{13}\cdot {\left(L_{c}^{6}L_{13}^{}\right)}^{\frac{N-3}{2}}\cdot L_{c}^{4}\cdot L_{30}^{}\cdot L_{tras}^{1},\;(x_{0}=1,y_{0}=N,x_{1}=M,y_{1}=1)\\ P_{in}^{\lambda_{s\_n}}\cdot L_{04}\cdot L_{c}^{4}\cdot {\left(L_{c}^{2}L_{42}\right)}^{\frac{M-3}{2}}\cdot L_{41}\cdot {\left(L_{c}^{6}L_{13}\right)}^{\frac{N-1}{2}}\cdot L_{c}^{2}\cdot L_{30}\cdot L_{tras}^{2},\;(x_{0}=1,y_{0}=N,x_{1}=M-1,y_{1}=1)\\ P_{in}^{\lambda_{s\_n}}\cdot L_{04}\cdot L_{c}^{4}\cdot {\left(L_{c}^{6}L_{42}\right)}^{\frac{M-3}{2}}\cdot L_{43}\cdot {\left(L_{c}^{6}L_{13}\right)}^{\frac{N-3}{2}}\cdot L_{c}^{4}\cdot L_{30}\cdot L_{tras}^{3},\;(x_{0}=1,y_{0}=N-1,x_{1}=M-1,y_{1}=1) \end{array}\right. \end{array}$$

(33) $$\begin{array}{l} P_{in}^{\lambda_{s\_n}}\cdot L_{\left(x_{0},y_{0}),(x_{1},y_{1}\right)}^{\lambda_{s\_n}}=\;(M\;is\;even,\;N\;is\;odd)\\ \left\{ \begin{array}{l} P_{in}^{\lambda_{s\_n}}\cdot {\left(L_{c}^{2}L_{42}^{}\right)}^{\frac{M-2}{2}}\cdot L_{41}^{}\cdot {\left(L_{c}^{2}L_{13}^{}\right)}^{\frac{N-1}{2}}\cdot L_{c}^{2}\cdot L_{30}^{}\cdot L_{tras}^{1},\;(x_{0}=1,y_{0}=N,x_{1}=M,y_{1}=1)\\ P_{in}^{\lambda_{s\_n}}\cdot L_{02}\cdot {\left(L_{c}^{6}L_{42}\right)}^{\frac{M-4}{2}}\cdot L_{c}^{6}\cdot L_{41}\cdot L_{c}^{2}\cdot {\left(L_{c}^{6}L_{13}\right)}^{\frac{N-1}{2}}\cdot L_{30}\cdot L_{tras}^{2},\;(x_{0}=1,y_{0}=N,x_{1}=M-1,y_{1}=1)\\ P_{in}^{\lambda_{s\_n}}\cdot L_{02}\cdot L_{c}^{6}\cdot {\left(L_{c}^{6}L_{42}\right)}^{\frac{M-4}{2}}\cdot L_{43}\cdot {\left(L_{c}^{6}L_{13}\right)}^{\frac{N-3}{2}}\cdot L_{c}^{4}\cdot L_{30}\cdot L_{tras}^{3},\;(x_{0}=1,y_{0}=N-1,x_{1}=M-1,y_{1}=1) \end{array}\right. \end{array}$$

(34) $$\begin{array}{l} P_{in}^{\lambda_{s\_n}}\cdot L_{\left(x_{0},y_{0}),(x_{1},y_{1}\right)}^{\lambda_{s\_n}}=\;(M\;is\;odd,\;N\;is\;even)\\ \left\{ \begin{array}{l} P_{in}^{\lambda_{s\_n}}\cdot L_{02}\cdot {\left(L_{c}^{6}L_{42}^{}\right)}^{\frac{M-3}{2}}\cdot L_{c}^{6}\cdot L_{41}^{}\cdot L_{c}^{4}\cdot {\left(L_{c}^{6}L_{13}^{}\right)}^{\frac{N-2}{2}}\cdot L_{10}^{}\cdot L_{tras}^{1},\;(x_{0}=1,y_{0}=N,x_{1}=M,y_{1}=1)\\ P_{in}^{\lambda_{s\_n}}\cdot L_{04}\cdot {\left(L_{c}^{2}L_{42}\right)}^{\frac{M-3}{2}}\cdot L_{c}^{4}\cdot L_{41}\cdot L_{c}^{4}\cdot {\left(L_{c}^{6}L_{13}\right)}^{\frac{N-2}{2}}\cdot L_{10}\cdot L_{tras}^{2},\;(x_{0}=1,y_{0}=N,x_{1}=M-1,y_{1}=1)\\ P_{in}^{\lambda_{s\_n}}\cdot L_{04}\cdot L_{c}^{4}\cdot {\left(L_{c}^{6}L_{42}\right)}^{\frac{M-3}{2}}\cdot L_{43}\cdot L_{c}^{6}\cdot {\left(L_{c}^{6}L_{13}\right)}^{\frac{N-4}{2}}\cdot L_{10}\cdot L_{tras}^{3},\;(x_{0}=1,y_{0}=N-1,x_{1}=M-1,y_{1}=1) \end{array}\right. \end{array}$$

In these formulas, $L_{trans}^{1}$, $L_{trans}^{2}$, and $L_{trans}^{3}$ represent the transmission loss of the first, second, and third longest optical links, respectively. $L_{ij}^{}$ represents the power loss when optical signal *λ_s_n_* travels from the injection port *i* of optical router R to its output port *j*.

Taking the first longest optical link (1, N) to (M, 1) as an example to analyze and compare the OSNR of different paths in detail, both M and N are even in this case. For path1, the OSNR of path1, the output power *P*_1_ at the destination router, and the power of the accumulated crosstalk noise *N*_1_ in path1 can be expressed as:

(35) $$OSNR_{1}=10\log(P_{1}/N_{1})$$

(36) $$P_{1}=L_{04}{\left(L_{42}L_{c}^{2}\right)}^{M-2/2}L_{41}{\left(L_{13}L_{c}^{2}\right)}^{N-2/2}L_{10}L_{tras}^{1}$$

(37) $$\begin{array}{ll} N_{1}= & N_{\left(1,N\right)}{\left(L_{42}L_{c}^{2}\right)}^{M-2/2}L_{41}{\left(L_{13}L_{c}^{2}\right)}^{N-2/2}L_{10}L_{tras}^{1}\\ & +N_{\left(2,N\right)}L_{41}{\left(L_{c}^{2}L_{13}\right)}^{N-2/2}L_{10}L_{c}^{2}L_{tras}^{\left(2,N-1\right)}\cdot \frac{1-{\left(L_{c}^{2}L_{42}L_{tras}^{\left(2,0\right)}\right)}^{M-2/2}}{1-L_{c}^{2}L_{42}L_{tras}^{\left(2,0\right)}}\\ & +N_{\left(M,N\right)}{\left(L_{c}^{2}L_{13}\right)}^{N-2/2}L_{10}L_{tras}^{\left(0,N-1\right)}\\ & +N_{\left(M,N-1\right)}L_{c}^{2}L_{10}L_{tras}^{\left(0,2\right)}\frac{1-{\left(L_{c}^{2}L_{13}L_{tras}^{\left(0,2\right)}\right)}^{N-2/2}}{1-L_{c}^{2}L_{13}L_{tras}^{\left(0,2\right)}}\\ & +N_{\left(M,1\right)}+N_{FWM}^{1} \end{array}$$

In Equations (36) and (37), $L_{trans}^{1}$ means transmission loss and $N_{FWM}^{1}$ denotes the FWM crosstalk noise power in the first longest link, and they can be expressed as

(38) $$L_{tras}^{\left(x,y\right)}=10^{-\frac{\alpha (x\cdot L_{wd}+y\cdot L_{ht})}{10}}$$

(39) $$N_{FWM}^{1}=\sum_{L_{1}}\sum_{\lambda_{p}}\sum_{\lambda_{s}}P_{DFWM}(\lambda_{p},\lambda_{s})$$

(40) $$L_{1}=(M-1)\cdot L_{wd}+(N-1)\cdot L_{ht}$$

Similarly, the OSNR of path4 can be calculated as follows:

(41) $$OSNR_{4}=10\log(P_{4}/N_{4})$$

(42) $$P_{4}=L_{02}{\left(L_{c}^{6}L_{42}\right)}^{M-2/2}L_{c}^{4}L_{23}{\left(L_{c}^{6}L_{13}\right)}^{N-2/2}L_{c}^{4}L_{30}L_{tras}^{1}$$

(43) $$\begin{array}{ll} N_{4}= & N_{\left(1,N\right)}L_{c}^{4}{\left(L_{42}L_{c}^{6}\right)}^{M-2/2}L_{23}{\left(L_{13}L_{c}^{6}\right)}^{N-2/2}L_{c}^{4}L_{30}L_{tras}^{1}\\ & +N_{\left(3,N\right)}L_{23}{\left(L_{c}^{6}L_{13}\right)}^{N-2/2}L_{30}L_{c}^{4}L_{tras}^{\left(1,N-1\right)}\cdot \frac{1-{\left(L_{c}^{6}L_{42}L_{tras}^{\left(2,0\right)}\right)}^{M-2/2}}{1-L_{c}^{6}L_{42}L_{tras}^{\left(2,0\right)}}\\ & +N_{\left(M,N\right)}{\left(L_{c}^{6}L_{13}\right)}^{N-2/2}L_{c}^{4}L_{30}L_{tras}^{\left(0,N-1\right)}\\ & +N_{\left(M,N-2\right)}L_{c}^{4}L_{30}L_{tras}^{\left(0,1\right)}\frac{1-{\left(L_{c}^{6}L_{13}L_{tras}^{\left(0,2\right)}\right)}^{N-2/2}}{1-L_{c}^{6}L_{13}L_{tras}^{\left(0,2\right)}}\\ & +N_{\left(M,1\right)}+N_{FWM}^{1} \end{array}$$

Based on the mentioned analyses, in order to simplify the equations we proposed, it can be assumed that the power loss between different input and output ports is identical in the optical router. Moreover, the crosstalk noise generated at optical routers located at the same status in different paths has tiny differences, and some of them are completely consistent. Therefore, in this case, the assumptions are made as follows:

(44) $$L_{ij}=L,\;(i,j=0,1,2,3,4,i\neq j)$$

(45) $$N_{\left(2,N\right)}=N_{\left(3,N\right)}\;,\;N_{\left(M,N-1\right)}=N_{\left(M,N-2\right)}$$

(46) $$\frac{P_{1}}{P_{4}}=\frac{L_{04}L_{41}L_{10}}{L_{02}L_{23}L_{30}L_{c}^{2(M+N)}}$$

(47) $$\frac{P_{1}/N_{1}}{P_{4}/N_{4}}=\frac{1}{L_{c}^{2(M+N)}}\cdot \frac{N_{4}}{N_{1}}>1\Rightarrow SNR_{1}>SNR_{4}$$

According to Formulas (36) and (42), we can easily conclude Formula (46). Based on our assumption, Formula (45) can be concluded according to Equations (37) and (43). Thus, through the calculation in (47), we can observe that the OSNR of path4 is smaller than that of path1. The remaining comparison of OSNR_1_ to OSNR_4_ follows the same pattern. OSNR_2_ and OSNR_3_ can be easily calculated, and it can be found that they are both smaller than OSNR_4_. The analyses for the first longest links of the other three types have the same results. Therefore, four links can be seen as the worst-case candidate links from the first longest optical links, and they are named interior links. Further, a minimum OSNR link should exist in the interior links, the second, and the third longest optical links.

## 3. Numerical Simulation

**Figure 6:**
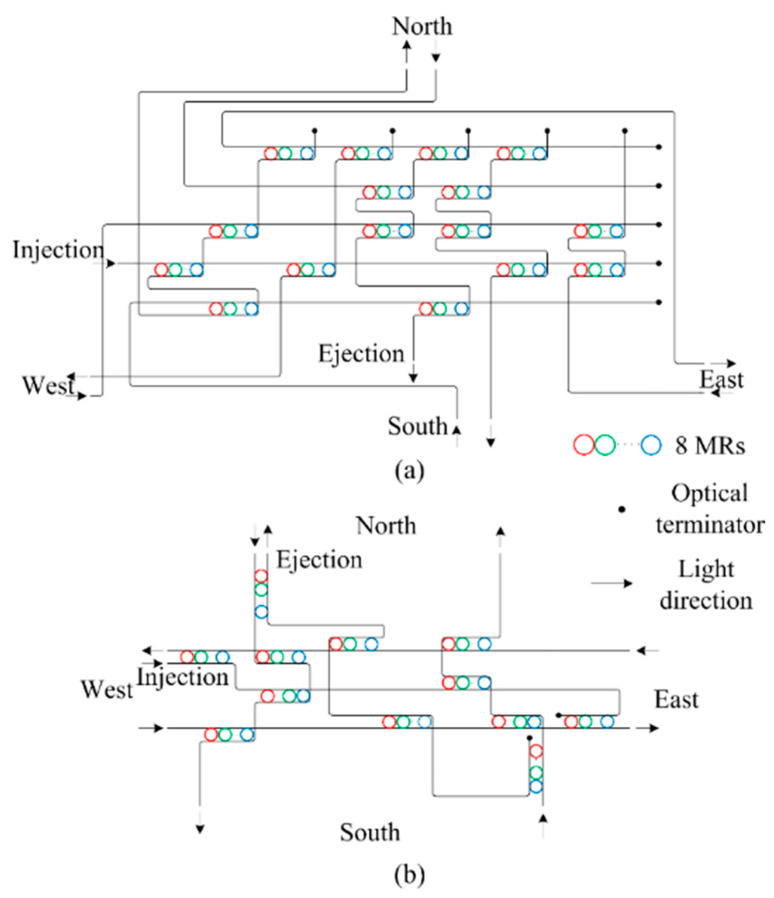
(**a**) WDM-based optimized crossbar (WOPC) optical router; (**b**) WDM-based crux (WCX) optical router.

Based on the basic analysis model, in this section, the performance of WDM-based torus ONoCs is evaluated in detail based on Matlab Matlab R2018a (developed by MathWorks, Inc., Natick, MA, USA), OptiSystem, and OPNET 14.5 (developed by Riverbed Technology, San Francisco, CA, USA). Matlab is used for the numerical simulation, OptiSystem is used to evaluate the transmission quality of optical signals in the WDM system, and the network throughput and latency indicators based on single-wavelength and WDM in 4 × 4 torus-based ONoCs can be obtained from the OPNET simulation. The wavelengths of 8-channel optical signals are selected from 1539.7 to 1545.3 nm with a 0.8 nm channel spacing, and the input optical power is 1 mW. The optical routers we selected for the simulation are the WDM-based optimized crossbar (WOPC) optical router and WDM-based crux (WCX) optical router, and the optical data follow the XY routing algorithm when it is transmitted in the routers. The architectures of the WOPC and WCX are shown in Figure 6. In the simulation, the size of the optical waveguide is 400 × 200 nm and the diameter of the MRs is around 10 µm. Moreover, the parameters of BOSEs can be acquired based on the finite-difference time-domain (FDTD) simulated results, which have been presented in detail in our previous work [16]. The remaining parameter values used in the simulation are shown in Table 1, Table 2 and Table 3.

### 3.1. The Nonlinear FWM Noise

**Figure 7:**
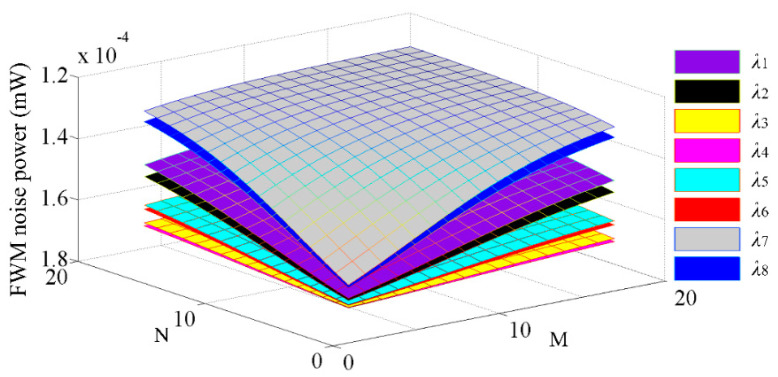
The FWM noise power at different wavelengths in M × N WDM-based torus ONoCs.

Based on the analysis of the FWM theoretical model, we next evaluate the effect of FWM noise on WDM-based torus ONoCs. As shown in Figure 7, which demonstrates the accumulated FWM crosstalk noise power at the destination IP core of the first longest links in M×N WDM-based torus ONoCs, the different colors represent the optical signals with different optical wavelengths. From the picture, we can draw a conclusion that the nonlinear crosstalk noise power introduced by the FWM effect decreases with increasing network scale and finally tends toward stability. Put another way, FWM nonlinear crosstalk noise decreases with the length of optical links. According to our analysis model, the power of crosstalk noise is proportional to FWM efficiency *η*. The FWM efficiency *η* decreases with the length of the optical transmission link due to increased phase mismatch between signals [44,45,46]. Moreover, the wavelengths located in the middle position will introduce higher FWM crosstalk noise power.

**Figure 8:**
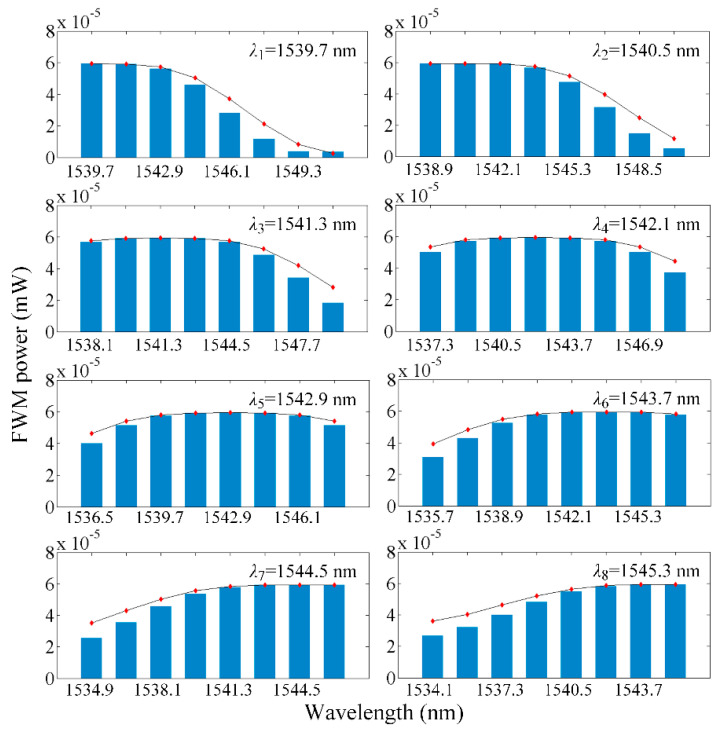
Newly generated FWM powers of eight optical wavelengths.

According to the FWM analysis model, the crosstalk noise power introduced by the FWM effect in the worst-case optical links can be calculated. One of the eight optical wavelengths can be seen as signal light and the others are pump lights. Thus, the interfering signals power in various wavelengths at the destination node can be seen in Figure 8. In Figure 8, the histogram represents the FWM crosstalk noise power at each wavelength in the first longest optical links when the network employs the WCX under the worst case, and the average power that accumulated on eight optical wavelengths is −37.67 dBm. The line chart stands for the FWM crosstalk noise in the first longest optical links when the network uses the WOPC under the worst case, and the average power is −37.60 dBm. It is noteworthy that if the length of different optical links is the same, the power of each newly generated optical wave is equivalent. Relevant parameters about the FWM are shown in Table 3.

### 3.2. OSNR and BER Evaluation

**Figure 9:**
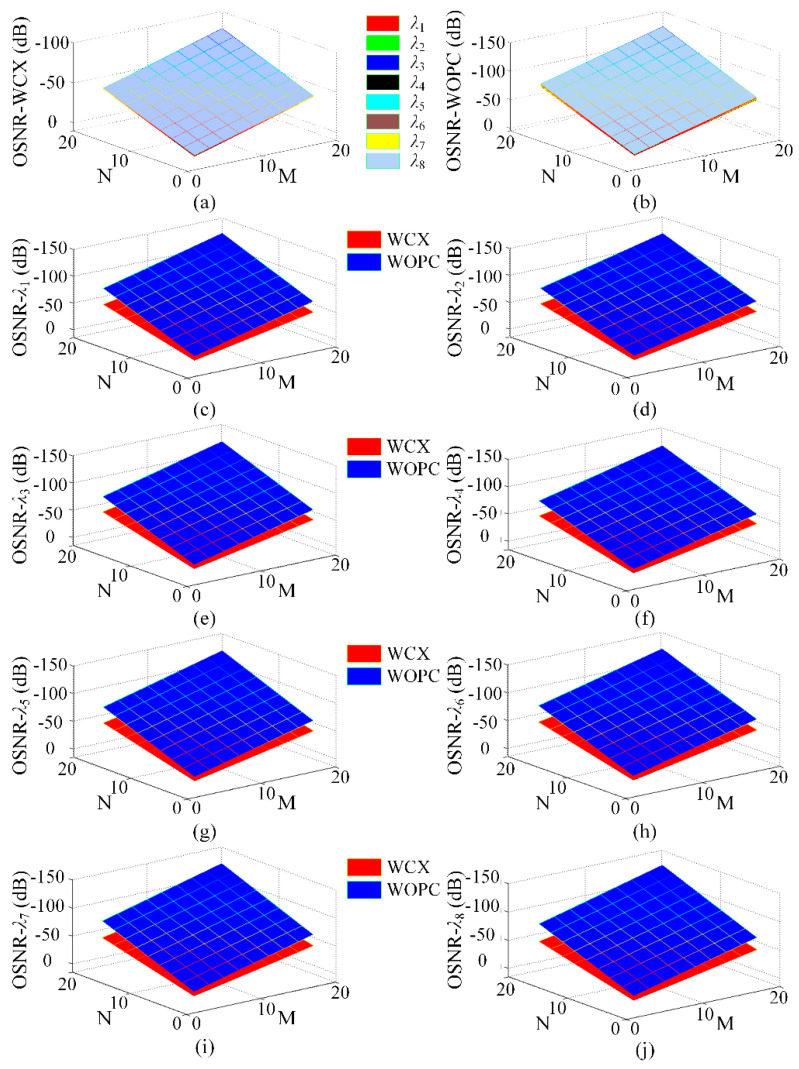
Optical signal-to-noise ratio (OSNR) comparison in M×N WDM-based torus ONoCs using the WOPC and WCX optical routers: (**a**,**b**) The OSNR at all wavelengths; (**c**–**j**) the OSNR at each wavelength.

To demonstrate the network performance when using WCX and WOPC optical routers under various network sizes, optical link (1, N) to (M, 1) where both M and N are even numbers is considered here. As shown in Figure 9, when M and N take different values, the change in OSNR of WDM-based torus ONoCs can be observed clearly. Obviously, as the network scale increases, the OSNR of each wavelength decreases significantly. In addition, the OSNR is different under the same network size when the different optical routers are adopted. From the comparison between Figure 9a,b, it is obvious that a better performance at the same network scale can be obtained when the WCX optical router is used.

In this paper, we consider that the value of the OSNR in the worst-case link greater than zero is the lower limit as the corresponding network scale is achievable. The numerical simulation results show that WDM-based torus ONoCs have a larger network scale when the WCX optical router is employed. The maximum network size is up to 6 × 5 and 5 × 6, and the corresponding links with the minimum OSNR are (M, N) to (1, 1) and (M, 1) to (1, N), respectively. Meanwhile, the network scale 5 × 4 can be obtained by using the WOPC optical router, and the link with the smallest OSNR is (1, 1) to (M, N). In the worst case, if the network size of WDM-based torus ONoCs with the WCX optical router is equal to or larger than 6 × 6, the optical signal power at the destination core is smaller than crosstalk noise power. Under the same condition, network size cannot be larger than 5 × 4 when using WOPC optical routers. Hence, the optimized optical devices and router structures are crucial factors in improving network performance.

**Figure 10:**
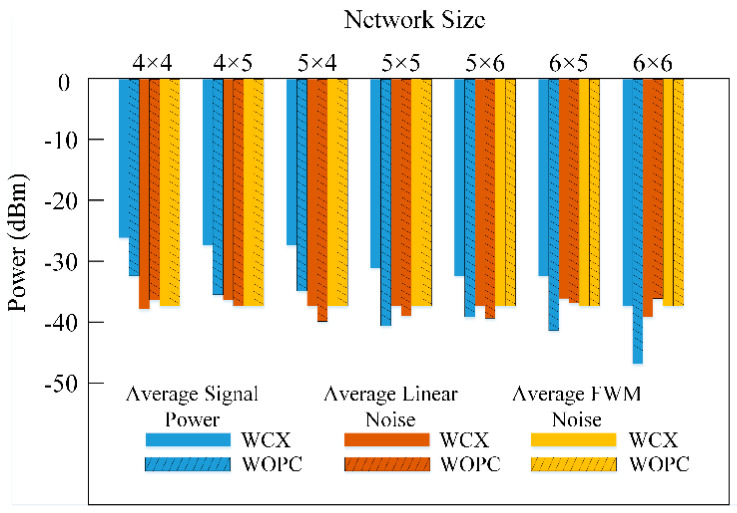
The comparison of average signal power, linear noise, and nonlinear FWM noise under different network sizes.

**Figure 11:**
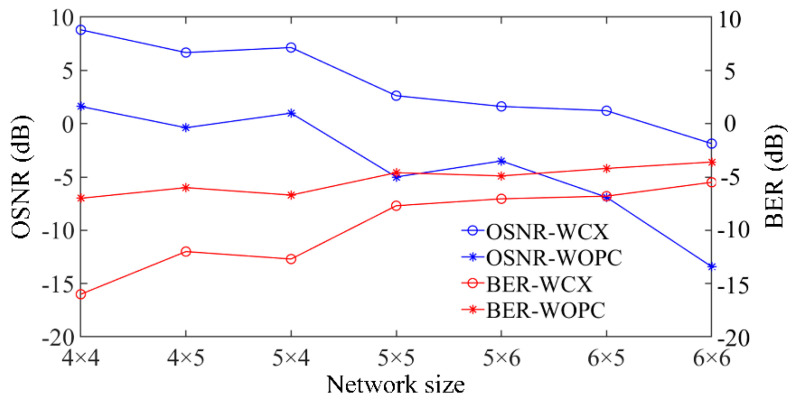
OSNR and BER comparison under different network sizes.

For the detailed comparison, the worst-case average signal power and linear and FWM nonlinear crosstalk noise powers in different network sizes are depicted in Figure 10. The comparisons of the average OSNR and BER of eight optical wavelengths between the WCX optical router and WOPC optical router are shown in Figure 11, in which we consider the input signals power as 1 mW. It can be seen that as the network scale increases, the worst-case signal power drops and finally falls below the crosstalk noise power. Furthermore, the FWM nonlinear noise also accounts for a large proportion of the total crosstalk noise power, and it is also a significant factor that influences network scalability and performance. With network size increasing, as shown in Figure 11, the OSNR declines and BER increases sharply. However, when using the WCX optical router, the network has better performance. For instance, when the scale of the WDM-based torus employing WCX optical routers is 4 × 4, the values of signal power, linear noise power, nonlinear FWM crosstalk noise power, and OSNR are −26.1 dBm, −38.4 dBm, −37.6 dBm, and 8.8 dB, respectively. However, if the WOPC optical router is used under the same network scale, the values are −32.3 dBm, −36.3 dBm, −37.6 dBm, and 1.6 dB, respectively.

**Figure 12:**
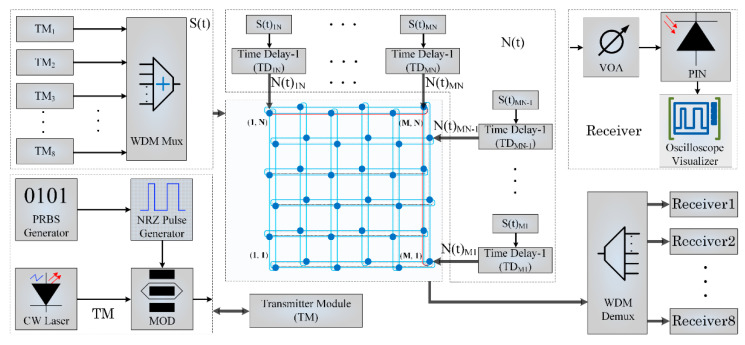
Diagram of transmission system of WDM-based torus ONoCs in OptiSystem.

In order to further evaluate the signal transmission quality, we set-up a multichannel optical communication system to simulate the performance of a 4 × 4 WDM-torus network. The basic components in the OptiSystem and experimental setup are introduced in [47,48]. The general system diagram is shown in Figure 12. We choose a continuous-wave laser as the light source whose wavelengths are from 1539.7 to 1545.3 nm with a wavelength interval of 0.8 nm; the power of each input optical signal is 1 mW. A pseudo-random bit sequence generator (PBRSG) has the same random characteristics with the information source of actual optical links, so it is used as the information source. The rate in the transmission system is 10 Gbit/s. Optical signals can be obtained by the modulation of the light source with the PBRSG and non-return-to-zero (NRZ) pulse generator, then eight optical signals are multiplexed into one channel by the optical multiplexer. Based on the analysis of the crosstalk noise model, crosstalk noise signals in the system can be seen as the optical signals with different time delays. According to the noise model presented in Section 2, sixteen equal optical signals with sixteen different time delays work as crosstalk noise signals. As shown in Figure 12, N(t) denotes the crosstalk noise signals and the right subscript in N(t) indicates which router introduces this crosstalk noise. Optical signals and crosstalk noise signals travel through the WDM-torus network and output signals connect with the PIN photodetector by the variable optical attenuator (VOA) and are converted into electrical signals. Some visualizers are linked with output ports to evaluate communication quality, and most of them are oscilloscopes that show the waveform of demodulated optical signals. The parameters settings in the transmission system are all based on the numerical simulation results in Matlab.

**Figure 13:**
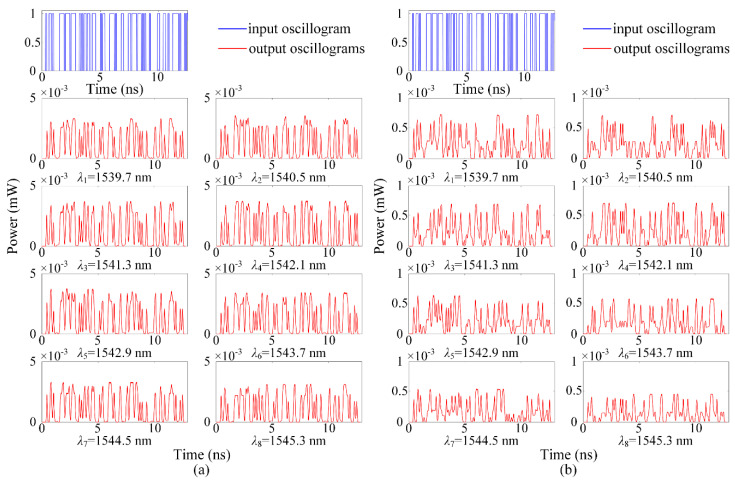
Signal input and output waveforms from $\lambda_{1}$ to $\lambda_{8}$: (**a**) Using WCX optical router; (**b**) using WOPC optical router.

Figure 13 shows the input and output signals when using WCX and WOPC optical routers, and it clearly indicates that the output signals at each wavelength have different levels of distortion and amplitude attenuation due to the effects of power loss and crosstalk noise. Figure 13a,b are the eight demodulated optical signals. The OSNR and crosstalk noise for each of the panel in Figure 13 are shown in Table 4. Compared with the waveform of the input signal, we can clearly see the power loss and distortion of the output optical signals. Furthermore, from the simulation results, we can find that the output signal has a higher power amplitude when using the WCX optical router, which completely corresponds to the simulation results in Matlab. Simulation results show that the power loss and crosstalk noise are both important factors that affect the performance of WDM-based ONoCs. Moreover, the choice of optical router is of great importance. The numbers of waveguide crossing, bending, and MRs should be taken into consideration in the design of an optical router to achieve a good performance and communication quality.

### 3.3. ETE-Delay and throughput Evaluation

**Figure 14:**
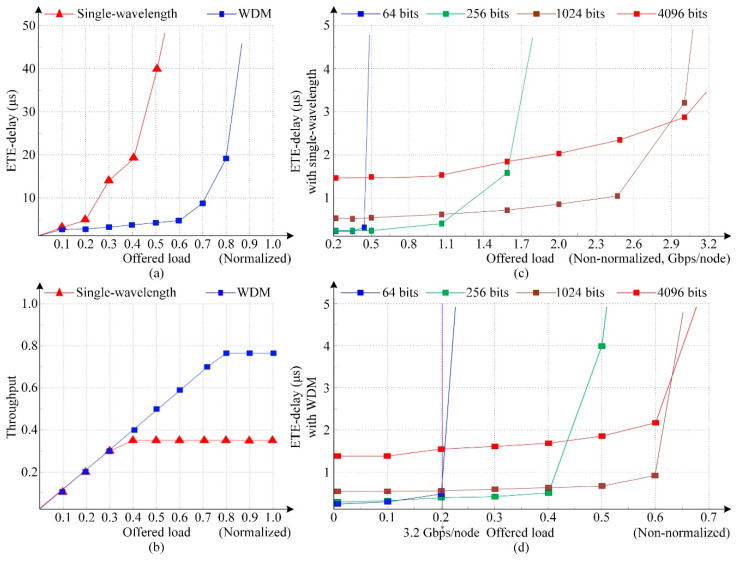
Network ETE delay and throughput comparison of torus-based ONoCs with single wavelength and WDM technology at data rate 12.5 Gbps.

To further evaluate the ETE-delay and throughput performance of torus-based ONoCs with single-wavelength and WDM technology, we set-up a 4 × 4 torus network model in OPNET. The transmission rate of the optical channel is 12.5 Gbps. The switch mechanism in our model is optical circuit switching (OSC) [49,50], in which optical data and control information are performed in the optical interconnection layer and electronic layer, respectively. The packet transmission in the simulation model follows the uniform traffic patterns. As shown in Figure 14a,b, the delay and throughput of a network using single-wavelength and WDM are compared with each other when the optical packet size is 1024 bytes. It can be seen that when the offered load is low, the ETE delay slowly increases as the offered load increases, but when the offered load exceeds a certain value, the network is congested until saturation, and the ETE delay increases sharply. Compared with the single-wavelength transmission network, the ETE delay of the WDM-torus ONoCs is relatively small within a wide range of offered load changes and also has a higher network saturation point.

Figure 14c,d demonstrate the trend of ETE delay in torus-based ONoCs with single-wavelength and WDM technology under different packet sizes, where packet sizes are adopted with 64, 256, 1024, and 4096 bits. As the packet length is small, the number of packets sent per unit time is large under the same offered load, so the data congestion is serious and the network reaches saturation first with sharply increased ETE delay. In addition, the saturation point of the network with large packet length is relatively high, and the delay will increase slowly. Furthermore, in the case of the WDM network, the ETE delay is greatly reduced compared to the network with single-wavelength and the throughput characteristics also have a great improvement.

**Figure 15:**
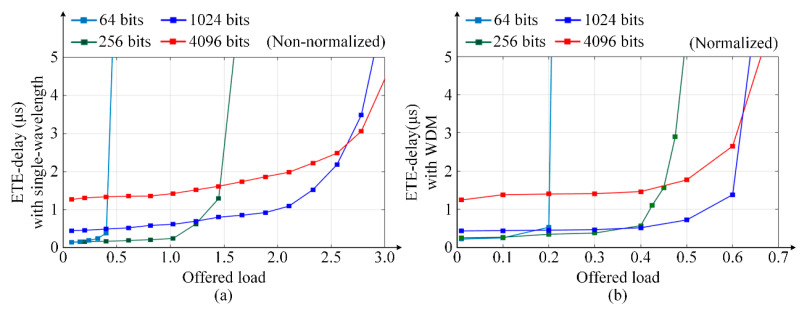
Network ETE delay and throughput comparison of torus-based ONoCs with single wavelength and WDM technology at data rate 40 Gbps.

Furthermore, the same simulation at higher data rate 40 Gbps is run to evaluate the impact of data rate on the performance of the torus-based ONoCs. The ETE delay of torus-based ONoCs with single wavelength and WDM technology at 40 Gbps is shown in Figure 15. Comparing the simulation results at 40 Gbps with the network ETE performance at 12.5 Gbps in Figure 14c,d, it can be concluded that the higher data rates will affect network performance slightly. For the same optical packet size, when the network has higher data rates, the time used to transmit payload packets will be reduced and the time interval between payload packets will be shorter. Then the more packets are sent per unit of time, it means that the packets have more competition for resources on network, resulting in the faster network saturation. On the other hand, if the offered load is small and the network resources are sufficient, the network will have a little better ETE delay performance with higher data rates.

## 4. Conclusions

Based on the torus topology, this paper proposes an insertion loss and crosstalk noise analysis model for WDM-based torus ONoCs from the bottom to upper layer. The general crosstalk noise and power loss model are hierarchically proposed at BOSEs and the optical router level; moreover, the OSNR and BER calculation methods are presented at the network level. The network performance research system is also established based on our simulation platform. WCX and WOPC optical routers are adopted in the numerical simulation to evaluate the network scalability, and the OSNR is calculated among the first, second, and third longest optical links to find the worst case. Simulation results indicate that both linear and nonlinear FWM crosstalk noise will restrict network scalability and influence network performance, and their power is very close under the worst case, which indicate that crosstalk generated by the FWM is non-negligible. The network can achieve a better performance when using the WCX optical router, and the same result can be observed in the simulation of OptiSystem. The maximum size of WDM-based torus ONoCs is 6 × 5 or 5 × 6 when the WCX optical router is employed, and it is 5 × 4 when the WOPC optical router is used. Moreover, the ETE delay and throughput characters are shown under different configurations. Compared with the single-wavelength network, they both have a great performance improvement in WDM-based torus ONoCs. Notably, the performance of WDM-based ONoCs can be further improved by the optimization of optical devices such as waveguide crossing angle optimization. The design of a new compact router is also another direction for the optimization. Moreover, preferable network structure and routing algorithms can both improve ONoC performance, and they are all worth studying in our further research.

## Figures and Tables

**Table 1:** Parameters of waveguide crossing and bending.

Notation	Parameter	Value	Reference
*K*_11_/*K*_12_	crossing crosstalk coefficient	−38.5 dB/90°	[35]
*L_c_*	crossing loss	−0.3358 dB/90°	[35]
*L_b_*	bending loss	−0.005 dB/90°	[42]

**Table 2:** Parameters of basic optical switching elements (BOSEs) [16].

Parameter Values (dB)	$K_{\lambda_{s\_n},off}^{\lambda_{s\_j}}$	$K_{\lambda_{s\_n},on}^{\lambda_{s\_j}}$	*Lp*_1_	*Lp*_2_
1539.7 nm (*λ*_1_)	−45.00	−19.25	−0.054	−1.101
1540.5 nm (*λ*_2_)	−44.75	−20.40	−0.045	−0.881
1541.3 nm (*λ*_3_)	−43.72	−21.88	−0.048	−0.715
1542.1 nm (*λ*_4_)	−43.01	−22.69	−0.064	−0.539
1542.9 nm (*λ*_5_)	−42.70	−23.38	−0.062	−0.532
1543.7 nm (*λ*_6_)	−41.73	−23.97	−0.083	−0.482
1544.5 nm (*λ*_7_)	−41.19	−24.74	−0.100	−0.358
1545.3 nm (*λ*_8_)	−39.53	−24.95	−0.148	−0.307

**Table 4:** OSNR and crosstalk noise for each of the panel in Figure 13.

Wavelength (nm)	Router	OSNR (dB)	Crosstalk Noise (dB)
1539.7 nm (*λ*_1_)	WCX	8.6928	−35.1203
	WOPC	1.4071	−34.1651
1540.5 nm (*λ*_2_)	WCX	8.9759	−35.0646
	WOPC	1.8280	−34.0215
1541.3 nm (*λ*_3_)	WCX	9.0935	−34.9527
	WOPC	2.0309	−33.8418
1542.1 nm (*λ*_4_)	WCX	9.2590	−34.8784
	WOPC	2.2826	−33.6940
1542.9 nm (*λ*_5_)	WCX	8.9439	−34.9272
	WOPC	1.8141	−33.8317
1543.7 nm (*λ*_6_)	WCX	8.6807	−34.8872
	WOPC	1.4417	−33.8315
1544.5 nm (*λ*_7_)	WCX	8.6269	−34.9601
	WOPC	1.3073	−33.9083
1545.3 nm (*λ*_8_)	WCX	8.0738	−34.8547
	WOPC	0.5264	−33.8736

**Table 3:** Parameters used in FWM noise model.

Notation	Parameter	Value	Reference
*α*	Attenuation coefficient	2.4 dB/cm	[37]
*A_eff_*	Effective waveguide core area	0.033 μm2	[37]
*λ*_0_	Zero dispersion wavelength	1555 nm	[43]
*n*_2_	Nonlinear refractive index	9 × 10^−18^ m^2^/w	[3]
*dD/dλ*	Chromatic dispersion slope	3.5 ps/nm^2^/km	[40]
